# Assessing the Clinical Appropriateness and Practical Utility of ChatGPT as an Educational Resource for Patients Considering Minimally Invasive Spine Surgery

**DOI:** 10.7759/cureus.71105

**Published:** 2024-10-08

**Authors:** Advith Sarikonda, Robert Abishek, Emily L Isch, Arbaz A Momin, Mitchell Self, Abhijeet Sambangi, Angeleah Carreras, Jack Jallo, Jim Harrop, Ahilan Sivaganesan

**Affiliations:** 1 Department of Neurological Surgery, Thomas Jefferson University, Philadelphia, USA; 2 Department of General Surgery, Division of Plastic Surgery, Thomas Jefferson University Hospital, Philadelphia, USA; 3 Department of Neurological Surgery, Thomas Jefferson University Hospital, Philadelphia, USA; 4 Department of Neurosurgery, Thomas Jefferson Medical College, Philadelphia, USA

**Keywords:** ai, chatgpt, minimally invasive spine surgery, patient education, readability

## Abstract

Introduction

Minimally invasive spine surgery (MISS) has evolved over the last three decades as a less invasive alternative to traditional spine surgery, offering benefits such as smaller incisions, faster recovery, and lower complication rates. With patients frequently seeking information about MISS online, the comprehensibility and accuracy of this information are crucial. Recent studies have shown that much of the online material regarding spine surgery exceeds the recommended readability levels, making it difficult for patients to understand. This study explores the clinical appropriateness and readability of responses generated by Chat Generative Pre-Trained Transformer (ChatGPT) to frequently asked questions (FAQs) about MISS.

Methods

A set of 15 FAQs was formulated based on clinical expertise and existing literature on MISS. Each question was independently inputted into ChatGPT five times, and the generated responses were evaluated by three neurosurgery attendings for clinical appropriateness. Appropriateness was judged based on accuracy, readability, and patient accessibility. Readability was assessed using seven standardized readability tests, including the Flesch-Kincaid Grade Level and Flesch Reading Ease (FRE) scores. Statistical analysis was performed to compare readability scores across preoperative, postoperative, and intraoperative/technical question categories.

Results

The mean readability scores for preoperative, postoperative, and intraoperative/technical questions were 15±2.8, 16±3, and 15.7±3.2, respectively, significantly exceeding the recommended sixth- to eighth-grade reading level for patient education (p=0.017). Differences in readability across individual questions were also statistically significant (p<0.001). All responses required a reading level above 11th grade, with a majority indicating college-level comprehension. Although preoperative and postoperative questions generally elicited clinically appropriate responses, 50% of intraoperative/technical questions yielded either "inappropriate" or "unreliable" responses, particularly for inquiries about radiation exposure and the use of lasers in MISS.

Conclusions

While ChatGPT is proficient in providing clinically appropriate responses to certain FAQs about MISS, it frequently produces responses that exceed the recommended readability level for patient education. This limitation suggests that its utility may be confined to highly educated patients, potentially exacerbating existing disparities in patient comprehension. Future AI-based patient education tools must prioritize clear and accessible communication, with oversight from medical professionals to ensure accuracy and appropriateness. Further research comparing ChatGPT's performance with other AI models could enhance its application in patient education across medical specialties.

## Introduction

Minimally invasive spine surgery (MISS) comprises a spectrum of techniques aimed at mitigating trauma and morbidity inherent in conventional open approaches to spinal procedures. Over the past three decades, MISS has undergone substantial evolution, finding application [1] in diverse spinal pathologies, including neural decompression and spinal fixation and fusion [2]. Noteworthy advantages of MISS over traditional spine surgeries encompass diminutive skin incisions, abbreviated hospital stays, expedited recovery periods, heightened postoperative pain relief, diminished blood loss, and a lower incidence of surgical site infections [3-6]. These merits render MISS particularly appealing to patients, who consistently perceive open spinal surgeries as more painful, expensive, sedative, and demanding lengthier recovery times relative to MISS alternatives [7,8].

In the digital age, patients are likely to explore the internet for information on MISS and potential candidacy for these procedures. Preoperative patient education, particularly in the context of spine surgeries, has proven to enhance patient satisfaction [9]. Agarwal et al. previously assessed the readability of information on various neurosurgery subspecialties extracted from the American Association of Neurological Surgeons website. Their study revealed that information related to MISS implicated a reading difficulty corresponding to a college or graduate level, significantly exceeding the reading level recommended by both the American Medical Association (AMA) at sixth grade [10] and the National Institutes of Health (NIH) at eighth grade [11]. Unfortunately, numerous online resources for patient education on spine surgery surpass the reading ability of the average American adult [12].

To address the complexity of existing online patient education resources, this paper explores the potential of Chat Generative Pre-Trained Transformer (ChatGPT), an artificial intelligence (AI) language model released in November 2022 with millions of monthly users [13]. Operating through a chatbox interface, ChatGPT responds adeptly to diverse user queries, including medical inquiries.

In the context of neurosurgery, ChatGPT has been posited as a learning tool for attendings and residents, as well as for patients. From a medical training perspective, ChatGPT has successfully passed standardized neurosurgical licensing exams, and it has also been used as a tool to generate literature reviews and individualized study materials [14]. From a patient standpoint, studies have examined the utility of ChatGPT as a "self-management tool" for patients with neurosurgical pathology, especially for those interested in or considering surgery [14]. Outside of neurosurgery, studies have examined ChatGPT's potential for patient education in various sectors, including cardiovascular disease, plastic surgery [15], and oncology [16], among others. In extending this exploration, we subjected ChatGPT to a series of frequently asked questions (FAQs) about MISS, derived from the literature (including the "frequently asked questions" section of the Society of Minimally Invasive Spine Surgery website [17]) and clinical expertise [18], adopting the patient's perspective.

While previous studies [19,20] have assessed the readability of online patient education resources for spine surgery, none have scrutinized both the clinical appropriateness and readability of ChatGPT's responses to MISS-specific questions. If ChatGPT proves capable of delivering appropriate and comprehensible responses on spine surgery, clinicians and hospital systems may consider replacing potentially intricate jargon currently present on their websites with ChatGPT-generated responses. Thus, our objective is to examine the clinical appropriateness and readability of these responses to ensure that patients interested in MISS access both clinically acceptable and understandable medical information.

## Materials and methods

We formulated a set of 15 FAQs concerning MISS. These questions were derived from the clinical expertise of the authors and were also adapted from literature assessing FAQs in MISS [17,21]. The categorization of these questions spanned three domains: preoperative, postoperative, and intraoperative/technical aspects. As an AI model, ChatGPT assimilates knowledge from user interactions, prompting us to input all questions independently into the software. Two questions, namely, "What are the various pain medications I have to take after MISS?" and "Will I become dependent on these pain medications?", were posed consecutively to ensure thematic cohesion. This approach aimed at preventing any bias or influence on ChatGPT-generated responses from prior questions. Additionally, each question was independently inputted into ChatGPT five times to assess response consistency. Responses to all questions are detailed in the Appendices.

To gauge the clinical appropriateness of ChatGPT's responses, we enlisted three neurosurgery attendings with expertise in MISS. Utilizing the rating protocol introduced by Sarraju et al. [18], reviewers evaluated sets of responses to five questions, categorizing them as "appropriate" or "inappropriate." Appropriateness is defined here in a patient-facing clinical context, assessing whether ChatGPT's responses were suitable for patient consumption on a hospital website. Appropriateness was a subjective combination of accuracy, readability, and accessibility. If a response entailed excessive technical jargon or conveyed incorrect information, surgeons marked the response as "inappropriate." An example of an "inappropriate" response, for example, would be a technically complicated answer to the simple question "Do you use lasers for MISS?". Reviewers also had the option to label a set of responses as "unreliable" if inconsistencies were identified between responses to the same question. For instance, if responses to the question "When should I consider consulting a spine surgeon?" were discordant with each other (with each response providing a markedly different indication for consulting a spine surgeon), this would be marked "unreliable."

**Figure 1:**
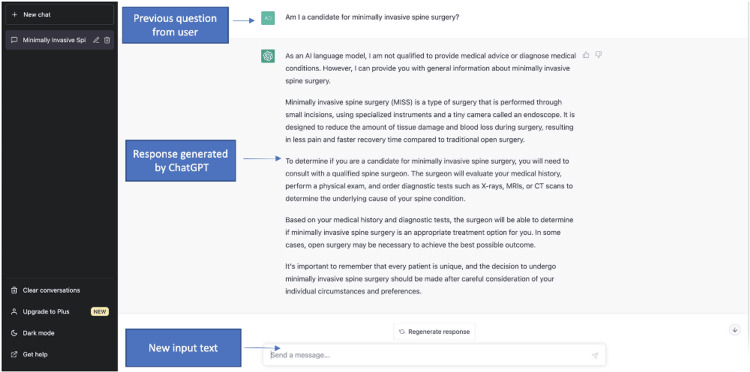
Example of a response to a frequently asked question generated by ChatGPT

The assessment of readability drew inspiration from previous studies employing a variety of readability assessment tools [17,20-22]. In our study, we employed seven widely recognized readability assessments. Six tests, namely, the Flesch-Kincaid (FK) Grade Level, Gunning Fog (GF) Index, Coleman-Liau (CL) Index, Simple Measure of Gobbledygook (SMOG) Index, Automated Readability (AR) Index, and FORCAST Grade Level test, provided scores indicative of the grade level required to comprehend the text excerpt. Simultaneously, the Flesch Reading Ease (FRE) test assigned a score on a 0-100 scale, with higher scores denoting easier readability [23]. We excluded readability formulas intended for foreign texts, as our responses were exclusively in English. An example of a standard input to ChatGPT is shown in Figure 1.

Descriptive statistics were performed using IBM SPSS Statistics for Windows, Version 29.0.2.0 (Released 2023; IBM Corp., Armonk, New York, United States). P-value was calculated using ANOVA analysis as appropriate to assess for differences in mean readability scores.

## Results

**Figure 2:**
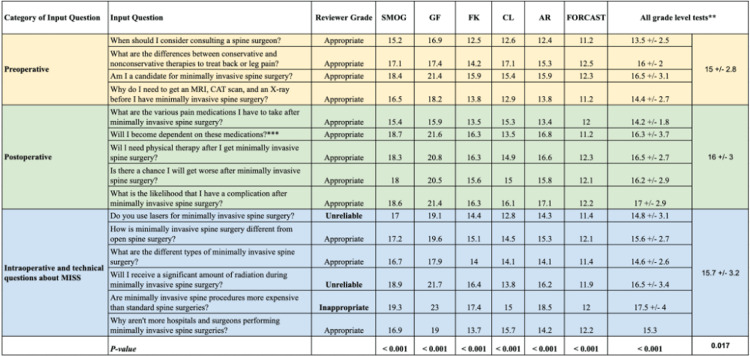
Mean readability scores and reviewer grades for each question *The Flesch Reading Ease assessment is not included in this table since its score does not correspond to grade-level difficulty.

The mean readability scores for responses to preoperative questions, postoperative questions, and intraoperative/technical questions were 15±2.8, 16±3, and 15.7±3.2, respectively. The mean readability scores for each question, divided among the various readability tests, are summarized in Figure 2. Differences in readability between these three groups were statistically significant (p=0.017), as were the differences in readability between each of our 15 questions (p<0.001), as expected considering the heterogeneity of these questions.

**Figure 3:**
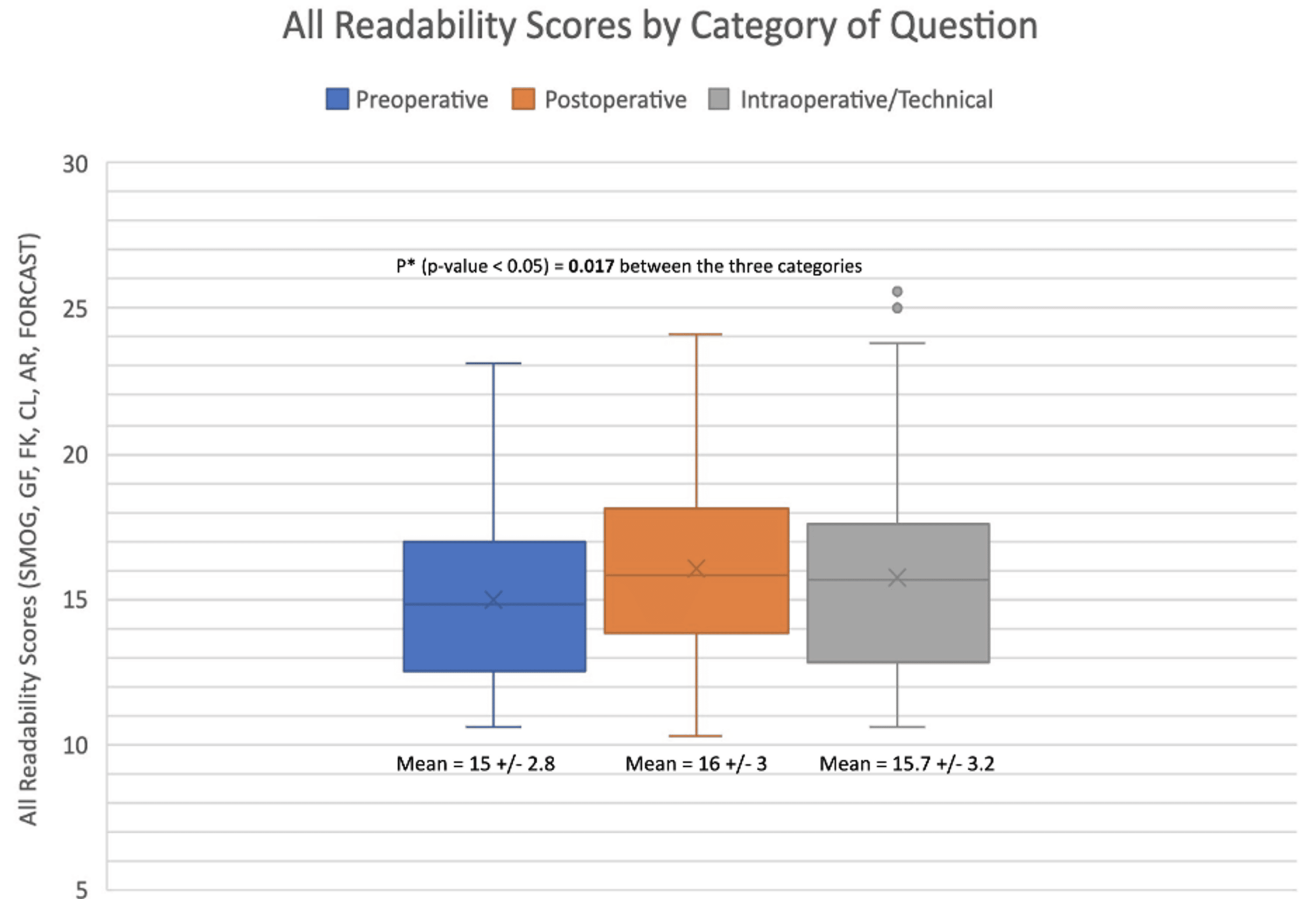
All readability scores sorted by category of question: mean score±standard deviation

In spite of these significant differences, though, all of ChatGPT's responses greatly exceeded the AMA and NIH's recommended grade-level readability of patient education resources. As indicated by the box-and-whisker plot in Figure 3, all of ChatGPT's responses were above an 11th-grade reading difficulty, with a majority of them implicating college-level reading difficulty. The mean reading grade levels for each of our questions ranged from 13.5 ("When should I consider consulting spine surgeons?") to 17.5 ("Are minimally invasive spine procedures more expensive than standard spine surgeries?"). This question regarding the cost of MISS had the largest standard deviation of any question (SD=4) and, interestingly, was the only question for whom ChatGPT's responses were deemed clinically "inappropriate."

Responses to preoperative and postoperative questions were all clinically appropriate, while three of the six (50%) intraoperative/technical questions were considered either clinically inappropriate or unreliable. "Will I receive a significant amount of radiation during MISS?" and "Do you use lasers for MISS?" both yielded "unreliable" responses due to response inconsistency, while responses to "Are minimally invasive spine procedures more expensive than standard spine surgeries?" were deemed inappropriate due to inaccuracy.

**Figure 4:**
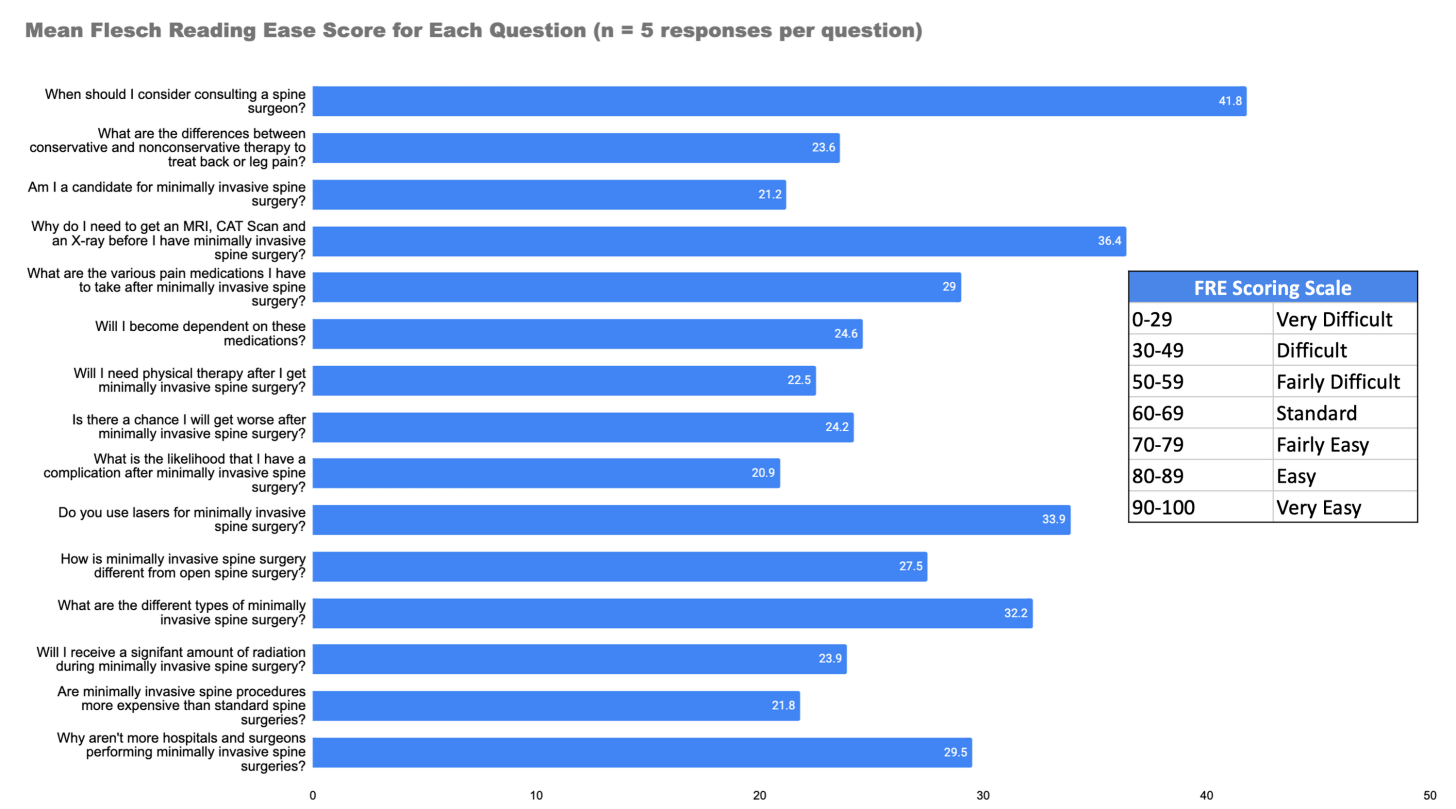
Mean FRE score for each question FRE: Flesch Reading Ease

FRE scores are summarized in Table 1 and Figure 4. The mean FRE scores ranged from 21 ("What is the likelihood that I will have a complication after MISS?" and "Am I a candidate for MISS?") to 36 ("Why do I need to get an MRI, a CT scan, and an X-ray before I have MISS?"), as displayed in Figure 4. It is worth noting that the question with the lowest mean readability score ("Am I a candidate for MISS?") also had the lowest FRE score, a contradiction that may be explained by the inherent differences in the formulaic composition of the FRE test compared to tests which provide readability grade-level estimates.

**Table 1:** Mean FRE scores, sorted by question and category FRE: Flesch Reading Ease; MISS: minimally invasive spine surgery

	Question	Mean FRE score per question (n=5 responses per question)	Mean FRE score per category	Difficulty
Preoperative questions	When should I consider consulting a spine surgeon?	41.8	31±10	Difficult to very difficult
	What are the differences between conservative and nonconservative therapy to treat back or leg pain?	23.6		
	Am I a candidate for MISS?	21.2		
	Why do I need to get an MRI, a CT scan, and an X-ray before I have MISS?	36.4		
Postoperative questions	What are the various pain medications I have to take after MISS?	29	24.2±6.9	Difficult to very difficult
	Will I become dependent on these medications?	24.6		
	Will I need physical therapy after I get MISS?	22.5		
	Is there a chance I will get worse after MISS?	24.2		
	What is the likelihood that I have a complication after MISS?	20.9		
Intraoperative and technical questions	Do you use lasers for MISS?	33.9	28.1±9	Difficult to very difficult
	How is MISS different from open spine surgery?	27.5		
	What are the different types of MISS?	32.2		
	Will I receive a significant amount of radiation during MISS?	23.9		
	Are minimally invasive spine procedures more expensive than standard spine surgeries?	21.8		
	Why aren't more hospitals and surgeons performing MISS?	29.5		
P-value		<0.001	0.045	

## Discussion

Preoperative patient education, especially in the context of spine surgery, has been associated with positive patient outcomes [24]. An integrated preoperative education model for spine surgery, for instance, demonstrated a reduction in patient anxiety and uncertainty both pre- and post-elective spine surgery [24]. However, not all patients receive adequate preoperative education, highlighting the necessity for accessible, clinically appropriate, and comprehensible online patient education resources.

From an appropriateness standpoint, ChatGPT exhibited proficiency in responding to preoperative and postoperative questions but faced challenges in generating appropriate and reliable responses to intraoperative and technical inquiries. Several factors may contribute to this discrepancy. Firstly, online information concerning intraoperative and technical aspects of MISS may be incongruent, limiting the utility of ChatGPT, which relies on textual data from the internet. Additionally, the nuanced nature of emerging fields like MISS may introduce disparities in information provided by surgeons, contributing to inconsistent responses. While our study attempted to mitigate subjectivity by excluding subjective questions, the inherent variability in medical practices and principles among surgeons introduces an element of inconsistency in responses to highly technical queries.

Despite these explanations for occasional inconsistencies in ChatGPT's responses, it's crucial to note that the software did not consistently produce appropriate answers. While ChatGPT holds promise in specific medical contexts, such as aiding in differential diagnosis [25], our findings underscore the importance of physician oversight to address its occasional inappropriate responses.

Beyond concerns about appropriateness, the complexity of ChatGPT-generated responses may limit its effectiveness for the average American adult patient. Our study's readability grade-level scores indicate that ChatGPT's educational utility may primarily benefit patients with a college- or graduate-level degree. Given the educational disparities in the United States, where a significant portion of the population has a high school diploma or less [26], expecting the average adult to comprehend the technical details of ChatGPT's responses, especially in the context of considering MISS, seems unrealistic. Education levels already disproportionately impact postoperative outcomes for spine surgery patients [27,28], and the use of complex language in online patient education resources, like ChatGPT, may exacerbate these disparities.

Additional applications of AI for neurosurgical patients

It is worth noting that the scope of AI in neurosurgery extends beyond chatbot-generated patient education. In addition to patient education, AI and machine learning models have been used for diagnostics, outcomes analysis, and treatment recommendations [29]. For example, well-trained AI models have outperformed traditional clinical approaches for brain tumor diagnoses, surgical risk stratifications, and tumor segmentation [29]. In this context, it is certainly possible that neurosurgeons may rely on AI models (including ChatGPT) to provide suggestions about the surgical approach that is most suitable for individual patients. This is especially important in elective spine surgery, a field that has notorious heterogeneity in indications and procedural approaches for treating pathology [30]. If a surgeon is unsure whether a MIS approach is better suited to address a patient's pathology compared to an "open" approach, the surgeon may incorporate AI into their surgical decision-making. Indeed, in a 2024 study, Sarikonda et al. tested the ability of chatbots to adhere to surgical guidelines for cervical fusion [31], which has diverse indications and utilization throughout the United States. AI has also been used to predict postoperative patient outcomes (based on patient-specific characteristics), including discharge disposition and functional improvement after surgery [29]. Patients who are therefore interested in MIS may utilize these AI applications to determine whether MIS is the right approach for them. Moreover, surgeons may utilize these tools in the clinic to help patients understand what they can realistically expect from surgery.

Limitations

This study has limitations, including the relatively small number of questions posed to ChatGPT compared to the myriad queries patients might have about MISS. Additionally, while readability assessments offer insights into reading grade-level difficulty, they become less informative beyond a certain threshold, making it challenging to objectively quantify response difficulty. The lack of a universally accepted readability test or a test with widespread applicability further complicates the objective assessment of response difficulty.

## Conclusions

Online patient education resources stand as valuable yet intricate tools for preoperative patient education. While ChatGPT holds potential in medical contexts, its application in patient education, particularly in the context of MISS, requires careful consideration of appropriateness, oversight by medical professionals, and acknowledgment of potential readability challenges for a diverse patient population. Even under such supervision, its utility may be limited to highly educated patients capable of comprehending its responses. Moving forward, the development of AI solutions for patient queries must prioritize conveying complex medical terminology in a manner accessible to the average American patient. Achieving this goal could enhance patient awareness and knowledge, potentially reducing adverse postoperative outcomes. In the interim, spine surgeons should actively explore avenues to educate their patients, and websites hosting patient education resources should strive to enhance content readability. Future research endeavors should investigate ChatGPT's role as a patient education tool in diverse surgical and medical domains. Additionally, comparative studies assessing ChatGPT's clinical appropriateness and readability against other leading interactive AI platforms could provide valuable insights for further refinement and optimization.

## Appendices

Q1: When should I consider consulting a spine surgeon?

Response 1

You should consider consulting a spine surgeon if you are experiencing persistent back pain or any other symptoms related to your spine, such as numbness or tingling in your arms or legs, weakness in your muscles, or difficulty controlling your bladder or bowels. Some specific situations where you may want to consult with a spine surgeon include the following: chronic back pain that has not improved with conservative treatments such as physical therapy, medication, or injections; symptoms such as numbness, tingling, or weakness that affect your arms or legs; sudden onset of severe back pain, especially if it is accompanied by weakness, numbness, or tingling in your arms or legs; a history of spinal injury or surgery that is causing ongoing pain or problems; persistent neck pain or stiffness that affects your ability to move your head or neck; scoliosis or other spinal deformities that are causing pain or difficulty with movement; and spinal tumors or infections.

If you are experiencing any of these symptoms or conditions, it is important to consult with a qualified spine surgeon who can evaluate your condition and recommend appropriate treatment options, which may include surgery or other interventions.

Response 2

You should consider consulting a spine surgeon if you are experiencing severe or persistent pain in your neck, back, or limbs that is affecting your quality of life and has not responded to non-surgical treatments such as physical therapy, medication, or injections.

Here are some specific signs and symptoms that may indicate a need for spine surgery:

Numbness or weakness in the arms or legs: This could be a sign of nerve compression, which may require surgery to relieve pressure on the affected nerve.

Loss of bladder or bowel control: This is a medical emergency and requires immediate evaluation by a spine surgeon.

Severe or worsening pain: If your pain is severe, chronic, or getting worse over time and is not responding to other treatments, it may be an indication that surgery is needed.

Spinal deformity: If you have a spinal deformity such as scoliosis, kyphosis, or a herniated disc that is causing pain or affecting your mobility, surgery may be necessary to correct the problem.

Trauma: If you have suffered a severe injury to your spine, such as a fracture or dislocation, you may need to see a spine surgeon for evaluation and possible surgical intervention.

It's important to remember that spine surgery is usually considered a last resort and that most back and neck problems can be treated without surgery. Your doctor can help you determine whether surgery is necessary and guide you through the decision-making process.

Response 3

Spine surgery is typically considered a last resort treatment option for back or neck pain and other spine-related conditions. Before considering spine surgery, you should first try non-surgical treatments such as physical therapy, medications, and other conservative treatments.

You may want to consider consulting a spine surgeon if you have the following: persistent and severe pain that does not improve with non-surgical treatments; weakness, numbness, or tingling in your arms or legs; loss of bowel or bladder control; spinal deformities or abnormalities, such as scoliosis or kyphosis; severe trauma or injury to your spine; and tumors or infections in your spine.

In general, it is a good idea to consult with a spine surgeon if you are experiencing significant pain or symptoms that are affecting your quality of life and you have not been able to find relief through conservative treatments. Your primary care physician or a specialist such as a neurologist or orthopedist can refer you to a spine surgeon for further evaluation and treatment recommendations.

Response 4

It's best to consult a spine surgeon when you have chronic or severe back pain, neck pain, or other spine-related problems that have not responded to non-surgical treatments. You should also consider seeing a spine surgeon if you experience any of the following:

Numbness or weakness in the arms or legs: If you have numbness or weakness in your arms or legs, it could be a sign of a spinal cord or nerve problem that requires immediate medical attention.

Loss of bowel or bladder control: If you experience sudden loss of bowel or bladder control, it could be a sign of a serious spinal condition and requires immediate medical attention.

Severe or worsening pain: If you experience severe or worsening pain, especially if it is accompanied by numbness or weakness, it could be a sign of a serious spinal problem.

History of spinal injury or surgery: If you have a history of spinal injury or surgery, you may need to consult a spine surgeon for ongoing evaluation and treatment.

Chronic back or neck pain: If you have chronic back or neck pain that has not responded to conservative treatments such as physical therapy, medications, or chiropractic care, it may be time to consider consulting a spine surgeon.

In general, it's important to consult with your primary care physician or a specialist if you experience any persistent or concerning symptoms related to your spine. They can help you determine whether a spine surgeon is the right choice for you and recommend the best course of treatment.

Response 5

If you're experiencing persistent or severe back pain or other symptoms such as weakness, numbness, or tingling in your arms or legs, you should consider consulting a spine surgeon.

Spine surgeons are specialized doctors who focus on treating conditions that affect the spine and related structures. They can evaluate your condition and recommend a range of treatments, including conservative measures such as physical therapy, medication, or injections, or more invasive options such as surgery.

In general, you may consider consulting a spine surgeon if you experience any of the following: chronic or severe back pain that hasn't improved with non-surgical treatments; numbness, tingling, or weakness in your arms or legs; difficulty walking or standing for extended periods; loss of bowel or bladder control; spinal deformity or curvature; and traumatic injury to the spine.

It's important to note that not all back problems require surgery and spine surgeons will often try non-surgical options first. However, if conservative treatments aren't effective, surgery may be necessary to relieve your symptoms and restore your quality of life.

Q2: What are the differences between conservative and nonconservative therapies to treat back or leg pain?

Response 1

Back or leg pain can be treated using different approaches, including conservative and nonconservative therapies.

Conservative therapies are typically non-invasive, are drug-free, and do not involve surgery. These therapies aim to alleviate pain and discomfort while promoting natural healing processes. Examples of conservative therapies for back or leg pain include:

Physical therapy: Exercises and stretches prescribed by a physical therapist to strengthen the muscles, improve flexibility, and reduce pain.

Chiropractic care: A treatment method that involves manual manipulation of the spine and other joints to improve alignment, relieve pain, and restore normal function.

Massage therapy: A hands-on approach that helps relax muscles, reduce tension, and improve blood flow to the affected area.

Acupuncture: A treatment method that involves inserting thin needles into specific points on the body to stimulate the nervous system and promote healing.

On the other hand, nonconservative therapies may involve invasive procedures, medications, or surgery. These therapies are usually recommended when conservative approaches have not been successful or when the condition is severe. Examples of nonconservative therapies for back or leg pain include:

Medications: Pain relievers, anti-inflammatory drugs, and muscle relaxants prescribed by a doctor to alleviate pain and discomfort.

Injections: Epidural steroid injections or nerve blocks to relieve inflammation and pain.

Surgery: Procedures such as spinal fusion, discectomy, or laminectomy to correct the underlying structural problems causing the pain.

Overall, conservative therapies are usually the first line of treatment for back or leg pain, while nonconservative therapies are reserved for more severe cases that do not respond to conservative approaches. It is important to consult a healthcare professional to determine the most appropriate treatment approach for your specific condition.

Response 2

Conservative therapies are non-invasive, non-surgical treatments that aim to manage back or leg pain by relieving symptoms and promoting healing. These treatments can include:

Physical therapy: Exercises, stretches, and other techniques to improve strength, flexibility, and range of motion in the spine and surrounding muscles.

Medications: Pain-relieving medications such as nonsteroidal anti-inflammatory drugs (NSAIDs), muscle relaxants, and opioids.

Chiropractic care: Manual adjustments to the spine and other joints to improve alignment and relieve pain.

Acupuncture: Insertion of thin needles into specific points on the body to stimulate healing and relieve pain.

Massage therapy: Manipulation of soft tissues in the back and surrounding areas to improve circulation, reduce inflammation, and relieve pain.

Nonconservative therapies are more invasive treatments that may involve surgery or other medical procedures to manage back or leg pain. These treatments can include:

Epidural steroid injections: Injection of a corticosteroid medication directly into the spinal canal to reduce inflammation and relieve pain.

Radiofrequency ablation: Destruction of nerve tissue in the spine using heat generated by radio waves to reduce pain signals.

Spinal decompression: A surgical procedure to relieve pressure on the spinal cord or nerves by removing a portion of the vertebrae or disc material.

Spinal fusion: A surgical procedure to permanently join two or more vertebrae together to stabilize the spine and reduce pain.

In general, conservative therapies are the first line of treatment for back or leg pain, and nonconservative therapies are considered when conservative treatments fail to provide adequate relief. However, the choice of treatment will depend on the specific cause of the pain, the severity of symptoms, and individual patient preferences and needs.

Response 3

Conservative therapies for back or leg pain include non-invasive treatments that do not involve surgery or other invasive procedures. These may include:

Rest and activity modification: This may involve limiting or avoiding certain activities that aggravate the pain while also incorporating gentle exercises and stretches.

Physical therapy: Physical therapy involves exercises, stretches, and other techniques to improve strength, flexibility, and mobility, reduce pain, and prevent future injuries.

Chiropractic care: Chiropractic care involves manual manipulation of the spine to improve alignment and alleviate pain.

Acupuncture: Acupuncture involves the insertion of needles into specific points of the body to relieve pain and improve function.

Massage therapy: Massage therapy involves manual manipulation of the soft tissues of the body to promote relaxation, reduce pain, and improve circulation.

Nonconservative therapies for back or leg pain include invasive procedures, such as surgery or injections, that may involve significant risks and side effects. Examples include:

Surgery: Surgery may be recommended in cases where conservative treatments have not been effective or where there is a significant structural issue that requires intervention.

Injections: Injections may be used to provide pain relief or reduce inflammation, but may also have side effects such as infection or nerve damage.

Nerve ablation: Nerve ablation involves the destruction of nerves that transmit pain signals, which may provide long-lasting pain relief, but also has risks such as nerve damage or infection.

Overall, conservative therapies are generally considered to be safer and less invasive than nonconservative therapies, but may also be less effective in some cases. It is important to consult with a healthcare professional to determine the best treatment approach for your specific condition.

Response 4

Conservative therapies are non-invasive or minimally invasive treatments that aim to alleviate back or leg pain without resorting to surgical intervention. Conservative therapies include physical therapy, exercise, chiropractic, acupuncture, massage therapy, and medication. These therapies are usually recommended as the first line of treatment for back or leg pain.

On the other hand, nonconservative therapies are more invasive and typically involve surgical intervention. Nonconservative therapies include spinal fusion, disc replacement, and laminectomy. These treatments are generally reserved for cases where conservative therapies have failed or when the pain is severe and debilitating.

The main difference between conservative and nonconservative therapies is that conservative therapies are less invasive and have fewer risks and side effects compared to nonconservative therapies. Conservative therapies also tend to focus on treating the underlying cause of the pain, whereas nonconservative therapies focus more on managing the symptoms of the pain.

Overall, the choice between conservative and nonconservative therapies will depend on the severity and underlying cause of the pain, as well as the patient's preferences and overall health status. In many cases, conservative therapies can effectively manage back or leg pain, allowing patients to avoid more invasive treatments.

Response 5

Back and leg pain can be treated with various conservative and nonconservative therapies. Conservative therapies are typically non-invasive and aim to relieve pain and inflammation without surgery. In contrast, nonconservative therapies are more invasive and often involve surgical intervention. Here are some of the main differences between conservative and nonconservative therapies for back and leg pain:

For conservative therapies, the following are some of the main differences:

Medications: Over-the-counter pain relievers such as acetaminophen or NSAIDs are usually the first line of defense for mild to moderate back or leg pain. Prescription medications, such as muscle relaxants or opioids, may be recommended for more severe pain.

Physical therapy: This involves exercises, stretching, and other techniques designed to strengthen the muscles and improve flexibility. Physical therapy may also include modalities such as heat or ice therapy, ultrasound, or electrical stimulation.

Chiropractic care: Chiropractors use manual manipulation to help relieve pain and improve spinal alignment.

Massage therapy: Massage therapists use various techniques to help reduce muscle tension, improve circulation, and promote relaxation.

Acupuncture: This involves the insertion of thin needles into specific points on the body to stimulate the body's natural healing processes.

For nonconservative therapies, the following are some of the main differences:

Surgery: This may be recommended if conservative therapies have failed to relieve pain or if there is a structural problem that requires correction, such as a herniated disc.

Injections: Steroid injections may be used to reduce inflammation and relieve pain.

Radiofrequency ablation: This involves the use of heat to destroy nerve tissue, which can help reduce pain signals.

Spinal cord stimulation: This involves the implantation of a small device that sends electrical signals to the spinal cord to block pain signals.

In general, conservative therapies are preferred for the initial treatment of back and leg pain, as they are less invasive and have fewer risks and complications. However, if conservative therapies are not effective, nonconservative therapies may be considered.

Q3: Am I a candidate for MISS?

Response 1

As an AI language model, I am not qualified to diagnose or provide medical advice. However, I can provide general information about MISS and the factors that may make someone a candidate for this type of procedure.

MISS is a type of surgical procedure that uses specialized instruments and techniques to perform spinal surgery with less damage to surrounding tissues than traditional open surgery. This type of surgery can be used to treat a variety of spinal conditions, including herniated discs, spinal stenosis, and spinal fractures.

Whether or not someone is a candidate for MISS depends on several factors, including:

The type and severity of the spinal condition: MISS may be an option for certain types of spinal conditions, such as herniated discs, but not for others, such as advanced spinal deformities.

The patient's overall health: Patients who are in good health are generally better candidates for MISS than those who have other health conditions that may increase the risk of complications.

The patient's medical history: Patients who have undergone previous spine surgeries or who have other medical conditions that may affect their recovery may not be good candidates for MISS.

The patient's preferences: Some patients may prefer minimally invasive surgery over traditional open surgery, but this will depend on the individual's specific circumstances.

It's important to note that the decision to undergo MISS should be made in consultation with a qualified healthcare professional who can evaluate your specific condition and recommend the most appropriate treatment option for you.

Response 2

As an AI language model, I don't have access to your medical history or the ability to perform a physical examination. Only a licensed healthcare professional can determine whether you are a candidate for MISS.

That being said, MISS is typically recommended for certain conditions, such as herniated discs, spinal stenosis, and degenerative disc disease, when conservative treatments have not been effective.

Candidates for MISS usually have a specific spine condition that can be treated through minimally invasive surgery; have failed to improve with conservative treatments like medications, physical therapy, and injections; have no significant medical conditions that would increase the risk of surgery; and are not significantly overweight or obese.

If you are experiencing back or leg pain and are considering MISS, it is important to consult with a licensed healthcare professional to determine whether this treatment option is right for you. They can evaluate your medical history, perform a physical examination, and recommend the best treatment approach for your specific condition.

Response 3

As an AI language model, I do not have access to your medical history, physical examination, or imaging reports, therefore, I cannot give you a definite answer to this question. However, in general, MISS may be an option for individuals who have certain conditions that cause spinal problems, such as herniated discs, spinal stenosis, spinal deformities, and some types of spinal tumors.

Candidates for MISS typically have the following characteristics: the spinal condition causing the pain or discomfort is clearly identifiable and localized; the symptoms have not responded to conservative treatments, such as physical therapy, medication, or injections; there are no significant medical conditions that may increase the risks of surgery, such as obesity or heart disease; and the individual is generally healthy and can tolerate general anesthesia.

However, the decision to undergo MISS should always be made after a thorough evaluation by a qualified spine specialist. The specialist can assess your condition, review your medical history and imaging studies, and recommend the most appropriate treatment plan based on your unique circumstances.

Response 4

As an AI language model, I cannot provide a definitive medical diagnosis or recommend a specific treatment. However, I can provide you with general information about MISS and who may be a candidate.

MISS is a type of procedure that involves making small incisions and using specialized tools to access the spine, as opposed to traditional open surgery that requires a larger incision. This type of surgery may be used to treat various spine conditions, including herniated discs, spinal stenosis, and degenerative disc disease.

Whether you are a candidate for MISS will depend on various factors, including your specific spine condition, your medical history, and your overall health. Your doctor will likely evaluate your condition and determine if MISS is appropriate for you.

Generally, candidates for MISS are individuals with spine conditions that can be treated using this technique and who are in overall good health. This may include individuals who have not responded to non-surgical treatments or who have significant pain or disability due to their spine condition.

If you are experiencing spine-related symptoms or have been diagnosed with a spine condition, it is important to consult with a qualified medical professional to discuss your treatment options, including the possibility of MISS.

Response 5

As an AI language model, I cannot provide a medical diagnosis or advice. However, I can provide you with some general information about MISS to help you determine if you might be a candidate for the procedure.

MISS is a type of surgery that uses specialized instruments and techniques to access the spine with less disruption to the surrounding tissues than traditional open surgery. This can lead to less pain, less scarring, and a faster recovery time for patients.

Whether or not you are a candidate for MISS will depend on several factors, including the specific condition you have, the severity of your symptoms, and your overall health. Some conditions that may be treated with MISS include herniated discs, spinal stenosis, and degenerative disc disease.

To determine if you are a candidate for MISS, you will need to consult with a qualified spine surgeon. They will evaluate your condition, perform any necessary imaging tests, and recommend a treatment plan that is tailored to your specific needs.

It's important to note that MISS is not always the best option for every patient and, in some cases, open surgery may be necessary. Your surgeon will be able to discuss the benefits and risks of each option with you and help you make an informed decision about your treatment.

Q4: How is MISS different from open spine surgery? 

Response 1

MISS is a type of surgical procedure that uses specialized instruments and techniques to perform spinal surgery with less damage to surrounding tissues than traditional open spine surgery. The main differences between MISS and open spine surgery are:

Size of the incision: In open spine surgery, a large incision is made to allow the surgeon to access the spine. In MISS, a smaller incision is made, typically less than an inch in length, to insert specialized instruments and cameras into the surgical site.

Tissue disruption: Open spine surgery requires more disruption of surrounding muscles, ligaments, and tissues, while MISS involves less tissue disruption. This means that patients who undergo MISS may experience less pain, have a shorter recovery time, and may be able to return to normal activities more quickly.

Visualization: In open spine surgery, the surgeon has a direct line of sight to the surgical site. In MISS, the surgeon uses specialized cameras and imaging technology to visualize the surgical site.

Intraoperative monitoring: In open spine surgery, the surgeon may use intraoperative monitoring techniques to monitor the patient's neurological function during the surgery. This may not be necessary in all cases of MISS.

Overall, MISS is designed to provide the same surgical benefits as open spine surgery while minimizing the damage to surrounding tissues and structures. The decision to undergo MISS versus open spine surgery will depend on the patient's specific condition and the surgeon's recommendation based on factors such as the complexity of the surgery, the patient's overall health, and the expected outcomes of the procedure.

Response 2

MISS and open spine surgery are two different surgical approaches used to treat spinal conditions. The main differences between these two procedures are:

Incision size: In open spine surgery, a larger incision is made to access the affected area of the spine, whereas in MISS, one or several smaller incisions are made. These smaller incisions allow the surgeon to use specialized instruments and cameras to access the spinal area, without having to cut through muscle and other tissues.

Muscle and tissue damage: In open spine surgery, there is typically more muscle and tissue damage due to the larger incision and the need to retract muscles to access the spine. In contrast, MISS requires less tissue damage and muscle retraction, resulting in less trauma to the surrounding tissues and faster recovery times.

Length of hospital stay: In open spine surgery, patients typically require a longer hospital stay and a longer recovery period due to the more invasive nature of the procedure. MISS, on the other hand, may allow patients to go home the same day or within a few days of the surgery, and recovery times are generally shorter.

Risks and complications: Both types of surgery carry risks and complications, but MISS is generally associated with fewer risks and complications, such as infection and blood loss.

Effectiveness: Both open spine surgery and MISS can be highly effective in treating spinal conditions. However, the choice of surgical approach will depend on the specific condition being treated, as well as other factors such as the patient's medical history and overall health.

In summary, MISS is less invasive and results in less tissue damage, shorter hospital stays, faster recovery, and fewer complications than open spine surgery. However, the decision to use either approach should be made after a thorough evaluation by a qualified spine surgeon, who can recommend the most appropriate treatment plan based on the patient's individual circumstances.

Response 3

MISS and open spine surgery are two different surgical techniques used to treat spinal conditions. The main differences between the two techniques are the size of the incision and the extent of tissue disruption.

Open spine surgery involves making a large incision through the skin and muscles of the back to access the affected area of the spine. This can cause significant tissue damage, blood loss, and prolonged recovery times. Open surgery is usually reserved for more complex or severe spinal conditions that cannot be treated with minimally invasive techniques.

On the other hand, MISS uses small incisions, specialized instruments, and imaging guidance to access the spine with minimal disruption to surrounding tissues. This approach can result in less blood loss, shorter hospital stays, and faster recovery times compared to traditional open surgery.

During MISS, a small tube called a tubular retractor is inserted through a small incision in the skin and muscles of the back. The tubular retractor creates a tunnel to the affected area of the spine, allowing the surgeon to access the spine with specialized instruments and cameras. MISS can be used to treat a variety of spinal conditions, including herniated discs, spinal stenosis, and spinal tumors.

In summary, MISS offers many benefits over traditional open spine surgery, including smaller incisions, less tissue damage, reduced blood loss, shorter hospital stays, and faster recovery times. However, not all spinal conditions are amenable to MISS, and the decision to use this technique depends on the specific needs of the patient and the complexity of the condition being treated.

Response 4

MISS is a type of surgical technique that uses smaller incisions and specialized instruments to access the spine, compared to traditional open spine surgery. Here are some of the key differences between the two approaches:

Incision size: In open spine surgery, a larger incision is made to allow the surgeon access to the spine. In contrast, in MISS, small incisions (often less than an inch in size) are made, which reduces the amount of tissue and muscle that needs to be cut and also helps to minimize scarring.

Muscle and tissue disruption: In open spine surgery, the surgeon may need to cut through muscle and other soft tissues to access the spine. This can cause damage to nearby structures and increase the risk of complications. In MISS, specialized instruments and techniques are used to avoid cutting through muscles and other tissues, which can help to reduce damage and speed up recovery.

Visualization: In open spine surgery, the surgeon has a clear, direct view of the surgical site. In MISS, the surgeon uses specialized instruments and imaging technology (such as fluoroscopy or endoscopy) to visualize the spine and guide the surgical tools.

Hospital stay and recovery: Open spine surgery usually requires a longer hospital stay and a longer recovery time compared to MISS. Patients who undergo MISS typically experience less pain and discomfort after surgery and can often return to normal activities more quickly.

It's worth noting that not all spine surgeries can be performed using a minimally invasive approach. The decision to use MISS versus open spine surgery depends on the specific condition being treated and the patient's individual circumstances. A spine surgeon can help determine which approach is best for each patient.

Response 5

MISS is a technique used to perform spinal surgeries with smaller incisions and less tissue disruption compared to traditional open spine surgery. Here are some of the key differences between the two procedures:

Incision size: One of the main differences between MISS and open spine surgery is the size of the incision. In MISS, surgeons make small incisions, usually less than an inch in length, whereas in open spine surgery, the incision is typically several inches long.

Soft tissue disruption: In MISS, surgeons use specialized instruments and techniques to minimize damage to surrounding muscles, ligaments, and other soft tissues. This can lead to less blood loss, reduced postoperative pain, and faster recovery times.

Visualization: In open spine surgery, surgeons have direct visualization of the surgical site, whereas in MISS, visualization is achieved using a microscope or endoscope. However, with advances in technology, visualization in MISS is improving and becoming more comparable to that of open spine surgery.

Length of hospital stay: Patients who undergo MISS often have shorter hospital stays than those who have open spine surgery. This is because MISS is typically associated with less postoperative pain, fewer complications, and faster recovery times.

Risks and complications: Like any surgical procedure, both MISS and open spine surgery carry risks and potential complications. However, because MISS is less invasive, it is generally associated with a lower risk of infection, bleeding, and other surgical complications.

Overall, MISS has become an increasingly popular option for spinal surgery due to its potential benefits, including smaller incisions, less tissue disruption, and faster recovery times. However, not all spinal conditions are suitable for MISS, and your surgeon will need to determine whether it is the best option for you based on your individual circumstances.

Q5: What are the different types of MISS?

Response 1

There are several types of MISS, each with its own techniques and applications. Some of the most common types of MISS include:

Microdiscectomy: This procedure involves removing a portion of a herniated disc that is pressing on a nerve root, which can cause pain and other symptoms.

Endoscopic discectomy: This procedure uses an endoscope, a small camera attached to a thin tube, to view and remove a portion of a herniated disc through a small incision.

Percutaneous vertebroplasty or kyphoplasty: These procedures involve the injection of bone cement into fractured vertebrae to stabilize the bone and relieve pain.

Lumbar laminectomy: This procedure involves removing a portion of the lamina, the bony arch that covers the spinal cord, to relieve pressure on the spinal cord and nerve roots.

Transforaminal lumbar interbody fusion (TLIF): This procedure involves fusing two vertebrae together to stabilize the spine and alleviate pain.

Anterior cervical discectomy and fusion (ACDF): This procedure involves removing a herniated disc in the neck and fusing two vertebrae together to stabilize the spine and alleviate pain.

Minimally invasive spinal decompression: This procedure involves removing a portion of the bone or tissue that is compressing the spinal cord or nerve roots to relieve pressure and alleviate pain.

The specific type of MISS recommended for a patient will depend on the nature and severity of the spinal condition and the patient's overall health. It is important to consult a qualified healthcare professional to determine the most appropriate treatment option for each individual case.

Response 2

There are several types of MISS that may be performed depending on the patient's condition and the location of the affected area of the spine. Some of the most common types of MISS include:

Microdiscectomy: This procedure is used to treat herniated discs in the lumbar spine. The surgeon makes a small incision and removes a portion of the damaged disc that is pressing on the spinal nerves.

Laminectomy: This procedure is used to treat spinal stenosis, which is a narrowing of the spinal canal that puts pressure on the spinal cord and nerves. The surgeon removes the lamina, a portion of the vertebra that covers the spinal canal, to create more space for the spinal cord and nerves.

Vertebroplasty and kyphoplasty: These procedures are used to treat compression fractures in the spine. The surgeon injects a special type of cement into the fractured vertebra to stabilize it and reduce pain.

Spinal fusion: This procedure is used to treat conditions such as degenerative disc disease, spondylolisthesis, and scoliosis. The surgeon fuses two or more vertebrae together using bone grafts and/or implants to stabilize the spine and reduce pain.

Disc replacement: This procedure is used to treat herniated discs in the cervical spine. The surgeon removes the damaged disc and replaces it with an artificial disc to maintain mobility and reduce pain.

It's worth noting that not all spinal conditions can be treated with minimally invasive techniques and, in some cases, traditional open surgery may be necessary. The specific type of surgery recommended will depend on the individual patient's condition and the surgeon's recommendation.

Response 3

MISS is a type of surgery that involves making small incisions in the skin rather than large incisions, which are used in traditional open surgery. The aim of MISS is to minimize the damage to the surrounding tissues and structures, reduce the risk of complications, and speed up recovery time.

There are several types of MISS, including:

Endoscopic spine surgery: This involves using a thin, flexible tube with a camera and light at the end (endoscope) to access and treat the spine through a small incision. The endoscope allows the surgeon to visualize the affected area and make precise incisions to remove damaged tissue or perform other procedures.

Microdiscectomy: This procedure is used to treat a herniated disc in the spine. It involves making a small incision and using a microscope to remove the damaged part of the disc that is pressing on a nerve.

Percutaneous vertebroplasty and kyphoplasty: These procedures are used to treat vertebral compression fractures caused by osteoporosis or other conditions. A small incision is made, and a special type of cement is injected into the fractured vertebra to stabilize it and relieve pain.

Minimally invasive spinal fusion: This involves fusing two or more vertebrae together to stabilize the spine and relieve pain. The procedure is performed through small incisions, and special instruments are used to place screws, rods, and other devices in the spine to hold it in place.

Nucleoplasty: This is a minimally invasive procedure used to treat disc herniation. A small incision is made, and a special device is used to remove a small amount of tissue from the damaged disc, relieving pressure on the nerve.

These are just a few examples of the different types of MISS. The specific type of surgery recommended will depend on the patient's individual condition and the severity of their symptoms.

Response 4

MISS is a type of surgical procedure used to treat spinal conditions with smaller incisions and less tissue disruption, resulting in faster recovery times and less postoperative pain compared to traditional open surgery. There are several types of MISS, including:

Microdiscectomy: A procedure to remove herniated or bulging discs in the spine through a small incision using a microscope and specialized instruments.

Endoscopic discectomy: Similar to microdiscectomy, an endoscope is used to visualize the spine and remove the herniated or bulging disc through a small incision.

Percutaneous discectomy: A minimally invasive procedure where a needle is inserted into the disc to remove the herniated material under fluoroscopic guidance.

Spinal fusion: A procedure that joins two or more vertebrae to stabilize the spine and relieve pain. This can be performed using minimally invasive techniques, such as percutaneous instrumentation and TLIF.

Vertebroplasty/kyphoplasty: Procedures used to treat vertebral compression fractures caused by osteoporosis or other conditions. Cement is injected into the fractured vertebra to stabilize it and relieve pain.

Minimally invasive laminectomy: A procedure used to treat spinal stenosis, where the lamina (back part of the vertebra) is removed to relieve pressure on the spinal cord or nerves.

Artificial disc replacement: A procedure where a damaged disc is replaced with an artificial one, maintaining spinal flexibility and reducing pain.

The specific type of MISS recommended will depend on the patient's individual condition and the location and severity of their spinal problem.

Response 5

MISS is a surgical technique that is designed to minimize the damage to the muscles and tissues surrounding the spine during surgery. There are several different types of MISS, including:

Microdiscectomy: This is a minimally invasive procedure that is used to treat a herniated disc. The surgeon makes a small incision in the back and uses a microscope to remove the damaged portion of the disc that is pressing on the nerve.

Endoscopic spine surgery: This is a minimally invasive procedure that uses an endoscope to visualize and treat the spine. The surgeon makes a small incision in the back and inserts a tube with a camera and light attached to it. This allows the surgeon to see the spine and perform the surgery without having to make a large incision.

Percutaneous vertebroplasty and kyphoplasty: These are minimally invasive procedures that are used to treat vertebral compression fractures. The surgeon inserts a small tube through a small incision in the skin and into the fractured vertebra. They then inject bone cement into the vertebra to stabilize it.

Minimally invasive lumbar fusion: This is a minimally invasive procedure that is used to treat conditions such as degenerative disc disease, spinal stenosis, and spondylolisthesis. The surgeon makes a small incision in the back and uses special instruments to fuse the affected vertebrae together.

Overall, the goal of MISS is to reduce the damage to the surrounding tissues and muscles, minimize blood loss, and speed up recovery times for patients.

Q6: Why do I need to get an MRI, a CT scan, and an X-ray before I have MISS?

Response 1

Imaging tests such as MRI, CT scan, and X-ray are often used to help diagnose the specific spinal condition that is causing pain or other symptoms. These tests provide detailed images of the spine and can help identify the location and extent of any abnormalities.

Before MISS, these imaging tests are typically performed to help the surgeon plan the surgery and to ensure that the procedure is appropriate for the patient's specific condition. The surgeon will use the images to locate the exact location of the spinal problem and to plan the surgical approach.

For example, the MRI or CT scan may be used to identify the precise location of a herniated disc or a bone spur that is compressing the spinal cord or nerve roots. This information is critical in determining the specific type of MISS that is required and where the surgeon needs to access the spine to perform the procedure.

In addition, imaging tests can also help the surgeon identify any potential risks associated with the surgery, such as nearby blood vessels or nerves that could be damaged during the procedure.

In summary, imaging tests such as MRI, CT scan, and X-ray are important tools in the diagnosis and planning of MISS. These tests help ensure that the surgery is appropriate for the patient's specific condition and help the surgeon plan the procedure to minimize risks and optimize outcomes.

Response 2

Before undergoing MISS, it is common for patients to undergo various diagnostic imaging tests, including MRI, CT scan, and X-ray. These tests are important to help the surgeon accurately diagnose the condition and determine the best approach for treatment.

Here are some of the reasons why these imaging tests are necessary before MISS:

MRI: An MRI is a non-invasive test that uses a powerful magnet and radio waves to create detailed images of the spine. It provides high-resolution images of the soft tissues of the spine, including the discs, nerves, and surrounding structures. MRI helps the surgeon to identify the precise location of the problem and determine the best approach for treatment.

CT scan: A CT scan is a diagnostic imaging test that uses X-rays and computer technology to create detailed images of the spine. It provides a 3D image of the spine, which helps the surgeon to visualize the problem in greater detail. A CT scan can also help to identify any abnormalities that may not be visible on an X-ray.

X-ray: X-rays are often the first imaging test performed to evaluate the spine. They provide a two-dimensional image of the spine and can help to identify fractures, dislocations, and other bony abnormalities. X-ray is also used to monitor the progress of the healing after surgery.

By combining the information obtained from these imaging tests, the surgeon can get a more comprehensive view of the spine and the underlying condition. This helps the surgeon plan the surgery, select the appropriate technique and equipment, and improve the overall outcome of the procedure. In addition, these tests can help to identify any potential risks or complications associated with the surgery, such as the presence of underlying medical conditions or other factors that may affect the patient's ability to tolerate the procedure.

Response 3

Before undergoing MISS, your doctor may recommend getting an MRI, a CT scan, or an X-ray to help diagnose the problem and plan the procedure. These imaging tests can provide detailed information about the structure of your spine and the condition that is causing your symptoms.

Here are some reasons why these imaging tests may be necessary:

Identifying the problem: Imaging tests can help your doctor identify the exact location and extent of the problem in your spine, such as a herniated disc or spinal stenosis. This information is essential for planning the surgical procedure and ensuring that the surgeon has a clear understanding of the specific problem that needs to be addressed.

Planning the procedure: Based on the imaging results, the surgeon can plan the most appropriate and effective surgical approach. This may involve using specialized tools and instruments to access the affected area with minimal damage to surrounding tissues.

Assessing risks: Imaging tests can help your doctor assess the risks associated with the procedure, such as the risk of nerve damage or bleeding.

Monitoring progress: After the surgery, follow-up imaging tests can help your doctor monitor your progress and ensure that the surgery was successful.

In summary, imaging tests are an essential part of the diagnostic process and can provide valuable information that helps ensure the success of MISS.

Response 4

Your doctor may recommend getting an MRI, a CT scan, and/or an X-ray before having MISS for a few reasons:

To determine the exact location and extent of the problem: These imaging tests can provide detailed information about the condition of your spine and the surrounding tissues. This information can help your surgeon plan the surgery more precisely.

To identify any potential risks: Certain conditions or factors may increase the risk of complications during or after surgery. Imaging tests can help identify these risks so that your surgeon can take steps to reduce them.

To rule out other conditions: Symptoms in the spine can be caused by a variety of conditions, including fractures, tumors, and infections. Imaging tests can help identify these conditions so that appropriate treatment can be given.

To ensure the procedure is appropriate: MISS may not be appropriate for everyone. Imaging tests can help your surgeon determine whether the procedure is appropriate for your particular condition.

In summary, imaging tests are an important part of the preoperative evaluation process for MISS. They provide valuable information that can help ensure a successful outcome and reduce the risk of complications.

Response 5

Before having MISS, your doctor may recommend getting an MRI, a CT scan, and an X-ray to help diagnose the source of your spinal problem and to help plan the surgical procedure.

An MRI uses a powerful magnet, radio waves, and a computer to produce detailed images of the body's structures. An MRI can provide your doctor with detailed images of your spine, including the soft tissues and nerves, which may not be visible on an X-ray.

A CT scan uses X-rays and computer technology to create detailed images of the body. A CT scan can provide your doctor with detailed images of the bones in your spine and the surrounding tissues.

An X-ray is a simple diagnostic test that uses radiation to produce images of the bones in your spine. X-rays can show your doctor the structure of your spine, such as whether there are any fractures or abnormalities.

By using these imaging tests, your doctor can get a better understanding of your spinal problem and plan the best surgical approach. The imaging tests can also help your doctor identify any potential risks or complications associated with the surgery.

Therefore, these tests are essential to ensure that your surgery is successful, safe, and effective.

Q7: What are the various pain medications I have to take after MISS?

Response 1

The type of pain medications you will need after MISS will depend on various factors, such as the extent of the surgery, your individual pain tolerance, and any underlying health conditions you may have.

Typically, after MISS, you will be prescribed pain medications to help manage your postoperative pain. These may include:

NSAIDs: NSAIDs such as ibuprofen and naproxen are often used to reduce inflammation and relieve pain.

Acetaminophen: Acetaminophen is an over-the-counter pain medication that can be used to reduce pain and fever.

Opioids: Opioids such as oxycodone, hydrocodone, and codeine may be prescribed for more severe pain. However, these medications carry the risk of addiction and other side effects, so they are usually used for a short period and under close supervision.

Muscle relaxants: Muscle relaxants such as cyclobenzaprine can be prescribed to help relieve muscle spasms and tightness.

It's important to follow your doctor's instructions regarding pain management after surgery and to report any side effects or concerns you may have.

Response 2

The specific pain medications prescribed after MISS may vary depending on the individual patient's needs and the specific surgical procedure performed. However, here are some common types of pain medications that may be prescribed:

NSAIDs: These medications help reduce pain and inflammation. Examples include ibuprofen, naproxen, and aspirin.

Acetaminophen: This medication can be effective in managing mild to moderate pain.

Opioids: These medications are powerful painkillers and are often prescribed for more severe pain. Examples include oxycodone, hydrocodone, and morphine. However, due to the risk of addiction and other side effects, opioids are typically used for a limited time and under close supervision.

Muscle relaxants: These medications help reduce muscle spasms, which can be a source of pain after spine surgery. Examples include cyclobenzaprine and methocarbamol.

Nerve pain medications: If you experience nerve pain after surgery, your doctor may prescribe medications such as gabapentin or pregabalin to help manage this type of pain.

It's important to follow your doctor's instructions for taking pain medications, including the dosage and frequency. In addition, you should always let your doctor know if you experience any side effects or have concerns about your medication regimen.

Response 3

The specific pain medications prescribed after MISS can vary depending on a number of factors including the type of surgery performed, the extent of the surgery, the patient's medical history, and their current medications. However, here are some common pain medications that may be prescribed:

Acetaminophen (Tylenol): This is a commonly used pain reliever that is available over-the-counter or in prescription strength. It is often used in combination with other pain medications to provide additional pain relief.

NSAIDs: Medications such as ibuprofen (Advil, Motrin) or naproxen (Aleve) can help reduce inflammation and relieve pain. NSAIDs can be purchased over-the-counter or prescribed by your doctor.

Opioids: Stronger pain medications such as oxycodone (Percocet), hydrocodone (Vicodin), or morphine may be prescribed for more severe pain. Opioids are effective pain relievers but can be addictive and have side effects such as constipation and drowsiness.

Muscle relaxants: Medications such as cyclobenzaprine (Flexeril) or methocarbamol (Robaxin) can help relieve muscle spasms and stiffness that may occur after surgery.

Nerve pain medications: Medications such as gabapentin (Neurontin) or pregabalin (Lyrica) may be prescribed if you experience nerve pain after surgery.

It is important to take these medications as prescribed by your doctor and to notify them if you experience any side effects or have concerns about their effectiveness.

Response 4

The specific pain medications prescribed after MISS may vary depending on factors such as the type and extent of surgery, your medical history, and your individual needs and response to pain. However, some common pain medications that may be prescribed after MISS include:

NSAIDs: These medications, such as ibuprofen, aspirin, or naproxen, can help relieve mild to moderate pain and reduce inflammation.

Acetaminophen: Acetaminophen (Tylenol) is a pain reliever that can be used alone or in combination with other medications to manage mild to moderate pain.

Opioid medications: These medications, such as oxycodone, hydrocodone, or codeine, are more powerful pain relievers and are typically reserved for moderate to severe pain that cannot be managed with other medications.

Muscle relaxants: These medications, such as cyclobenzaprine or baclofen, can help relieve muscle spasms or tension that may contribute to pain after surgery.

It is important to follow your doctor's instructions for taking these medications, including the dosage and frequency of administration. You should also be aware of the potential side effects of these medications, such as dizziness, drowsiness, constipation, and nausea. If you have concerns or questions about your pain medication regimen after MISS, be sure to discuss them with your doctor or healthcare provider.

Response 5

As an AI language model, I cannot provide specific medical advice. However, I can give you general information about pain management after MISS.

After MISS, your doctor may prescribe one or more pain medications to manage your pain. These may include:

NSAIDs: NSAIDs, such as ibuprofen and naproxen, can reduce pain and inflammation. They may be prescribed for mild to moderate pain.

Acetaminophen: Acetaminophen (Tylenol) can help relieve pain, but it does not reduce inflammation. It may be prescribed for mild to moderate pain.

Opioids: Opioids, such as oxycodone and hydrocodone, can help relieve severe pain. However, they have a high risk of addiction and other side effects, so they are typically prescribed for short-term use.

Muscle relaxants: Muscle relaxants, such as cyclobenzaprine and baclofen, can help relieve muscle spasms that may occur after spine surgery.

Your doctor will determine the most appropriate pain management plan for you based on your individual needs and medical history. It's important to follow your doctor's instructions and never take more medication than prescribed.

Q8: Will I become dependent on these pain medications? (asked back to back/consecutively with the previous question)

Response 1

There is a risk of becoming dependent on pain medications if they are used for an extended period or if they are not taken as directed by your doctor. However, the risk of becoming dependent on pain medications after MISS is relatively low if you take the medications as prescribed and only for a short period of time.

It's important to discuss any concerns you may have about pain medication dependence with your doctor before and after surgery. Your doctor can help you understand the risks and benefits of different pain management options and develop a plan that minimizes the risk of dependence.

Additionally, if you are concerned about becoming dependent on pain medications, there are other pain management options that you can explore with your doctor, such as physical therapy, acupuncture, or mindfulness-based stress reduction techniques. These alternative therapies may be effective in managing pain and reducing the need for medication.

Response 2

It is possible to become dependent on some of the pain medications used after MISS, particularly opioids. However, the risk of developing a dependence on these medications can be minimized by using them only as prescribed and for a limited amount of time. Your doctor will work with you to create a pain management plan that balances the need for pain relief with the risk of dependence.

To reduce the risk of dependence on opioids, your doctor may gradually reduce the dosage of the medication or switch to a non-opioid pain reliever as your pain improves. They may also recommend other pain management techniques, such as physical therapy, acupuncture, or relaxation techniques, to help manage your pain without relying solely on medication.

It is important to communicate with your doctor about any concerns you may have regarding dependence on pain medications. Your doctor can help you understand the risks and benefits of these medications and work with you to create a personalized pain management plan that meets your individual needs.

Response 3

The risk of becoming dependent on pain medications after MISS depends on several factors, including the type and duration of the medication, your individual medical history and risk factors, and how closely you follow your doctor's instructions for use.

It is important to note that the use of opioids, which are commonly prescribed for pain after surgery, carries a risk of dependence and addiction. However, this risk can be reduced by following your doctor's instructions carefully, taking the medication only as prescribed, and gradually tapering off the medication as your pain improves.

Your doctor will work with you to develop a pain management plan that is tailored to your individual needs and will monitor your use of pain medications to ensure that you are using them safely and effectively. If you have a history of substance abuse or other risk factors for addiction, your doctor may recommend alternative pain management strategies or refer you to a specialist for additional support.

Response 4

There is a risk of becoming dependent on pain medications after MISS, especially if they are not used as prescribed or used for a long time. However, the risk of dependence can vary depending on factors such as the type of medication prescribed, the dose, and the duration of use.

In general, after MISS, most patients experience some pain and discomfort for a short period of time, typically a few weeks to a few months. Pain medications are often prescribed to manage this pain during the recovery period, and the goal is to gradually taper off these medications as the pain subsides.

To minimize the risk of dependence on pain medications after MISS, it's important to follow your doctor's instructions for taking the medication and to use it only for as long as necessary. Your doctor may recommend alternative pain management strategies, such as physical therapy or non-medication treatments, to help manage your pain during the recovery period.

If you are concerned about the risk of dependence on pain medications after MISS, talk to your doctor about your concerns and any alternative pain management strategies that may be available. Your doctor can work with you to develop a pain management plan that is tailored to your individual needs and helps to minimize the risk of dependence on medication.

Response 5

There is a risk of developing dependence on some of the pain medications used after MISS, particularly opioid medications. However, the risk of dependence varies depending on the type of medication, dosage, duration of use, and individual factors such as medical history and genetics.

To minimize the risk of dependence, your doctor may prescribe pain medications for a limited time, gradually taper the dosage as you recover, and recommend non-pharmacological pain management strategies such as physical therapy, acupuncture, or relaxation techniques.

If you have a history of substance abuse or addiction, it is important to discuss this with your doctor before taking any pain medications after MISS. Your doctor can work with you to develop a pain management plan that minimizes the risk of addiction and ensures your safety and comfort during recovery.

Q9: Will I need physical therapy after I get MISS?

Response 1

Yes, you may need physical therapy after MISS. Physical therapy can be an important part of your recovery process and can help you regain strength, flexibility, and mobility.

After MISS, your doctor may recommend physical therapy to help you:

Reduce pain: Physical therapy can help reduce pain and inflammation by improving circulation, promoting healing, and reducing muscle tension.

Regain mobility: Physical therapy can help you regain mobility and function by strengthening the muscles that support your spine and improving your range of motion.

Prevent complications: Physical therapy can help prevent complications such as blood clots and pneumonia by encouraging early mobilization and promoting deep breathing exercises.

Your physical therapy program will be tailored to your specific needs and may include exercises to strengthen your back and core muscles, stretches to improve your flexibility, and manual therapy techniques to reduce pain and improve mobility.

It's important to follow your physical therapy program closely and to communicate any concerns or issues with your therapist or doctor. With proper rehabilitation and a gradual return to activity, most patients can expect to make a full recovery after MISS.

Response 2

In many cases, physical therapy is an important part of the recovery process after MISS. The specific type and duration of physical therapy you may need will depend on a variety of factors, including the type of surgery you undergo, your age and overall health, and your individual needs and goals for recovery.

Physical therapy can help improve strength, flexibility, and range of motion in the spine and surrounding muscles, which can help reduce pain and improve function. Physical therapy may include exercises, stretches, and other interventions designed to promote healing and improve mobility.

Your doctor will work with you to develop a postoperative recovery plan that is tailored to your individual needs and may include physical therapy, along with other interventions such as pain management, medication management, and lifestyle changes. It is important to follow your doctor's instructions carefully and attend all recommended follow-up appointments to ensure the safest and most effective recovery possible.

Response 3

Physical therapy is often recommended after MISS to help you regain strength, mobility, and function. The specific type and duration of physical therapy will depend on the nature and extent of the surgery, as well as the patient's individual needs and recovery progress.

Physical therapy may include exercises to improve range of motion, flexibility, and strength, as well as techniques to reduce pain and swelling. Your physical therapist may also teach you how to perform activities of daily living safely and correctly, as well as provide advice on posture, body mechanics, and ergonomics.

In general, the goal of physical therapy after MISS is to help you return to your normal activities as quickly and safely as possible. By following your physical therapist's recommendations and actively participating in your rehabilitation program, you can improve your chances of achieving a successful outcome from your surgery.

Response 4

It is possible that you may need physical therapy after MISS, but it will depend on the specific details of your procedure and your individual circumstances.

MISS is a type of procedure that uses small incisions and specialized tools to access the spine, which can help reduce tissue damage, scarring, and recovery time compared to traditional open surgery. However, even with a minimally invasive approach, your spine and surrounding muscles may still experience some trauma and inflammation, which can cause pain and limit your range of motion.

Physical therapy can be helpful in managing these symptoms and restoring function after spine surgery. A physical therapist can work with you to develop a customized exercise and stretching program that targets the muscles and tissues affected by your surgery. They can also provide guidance on how to safely move and perform daily activities while your spine heals.

Ultimately, whether or not you need physical therapy after MISS will depend on a variety of factors, including the type and extent of your surgery, your age and overall health, and your goals for recovery. Your surgeon and other members of your healthcare team can help you determine whether physical therapy is right for you and what kind of program may be most beneficial.

Response 5

Whether or not you will need physical therapy after MISS will depend on several factors, including the specific type of surgery you had, your overall health and fitness level, and your doctor's recommendations.

In many cases, physical therapy can be helpful after MISS to help improve mobility and strength, reduce pain and inflammation, and prevent future injury or complications. Your doctor may recommend a specific physical therapy program tailored to your needs and goals.

However, it's important to note that every patient is unique and the need for physical therapy after surgery may vary. Your doctor will be able to give you more information about whether or not physical therapy is recommended for your specific case.

Q10: Do you use lasers for MISS?

Response 1

As an AI language model, I don't perform medical procedures or surgeries. However, I can provide general information about the use of lasers in MISS.

Lasers can be used in MISS procedures to remove or ablate tissue, such as herniated discs or bone spurs, with minimal damage to surrounding tissues.

Response 2

Although lasers are sometimes used in other types of surgery, such as eye surgery and skin surgery, they are not typically used in MISS.

The most common types of MISS use specialized instruments and imaging techniques to access and repair the spine. These techniques include small incisions, tubular retractors, and specialized surgical instruments such as endoscopes, microscopes, and laparoscopic instruments. The surgeon uses these tools to access the spine and perform the necessary repairs, such as removing a herniated disc or stabilizing the spine with screws and rods.

While lasers may be used in some very specific cases of spine surgery, such as in the treatment of tumors or certain nerve conditions, they are not widely used in MISS. This is because the laser's high energy can cause damage to nearby tissues and the precision of a laser is not always necessary for most spinal conditions. Your surgeon will discuss the appropriate surgical technique for your individual needs based on your diagnosis and the specifics of your case.

Response 3

Yes, lasers can be used in MISS, although they are not always a standard part of the procedure. Laser technology can be used to perform certain types of MISS, such as laser discectomy, in which a small laser is used to remove portions of a herniated disc.

However, it's important to note that while lasers can be an effective tool in some cases, they are not always necessary or appropriate for every patient or condition. The decision to use a laser in a minimally invasive spine procedure is typically made by the surgeon based on the specific needs of the patient and the goals of the surgery.

It's also important to understand that the use of a laser in spine surgery does not necessarily make the procedure less invasive. MISS is defined by the size and location of the incision and the extent of tissue disruption, not by the specific tools or technologies used. So while lasers can be a useful tool in some cases, they are not the defining characteristic of MISS.

Response 4

As an AI language model, I do not perform surgeries or use lasers. However, I can provide information on the use of lasers in MISS.

Laser technology has been utilized in MISS for the treatment of a variety of spine conditions. Laser spine surgery involves using a laser to remove or vaporize small amounts of tissue in the spine, with the aim of reducing pain and improving mobility.

One common use of lasers in MISS is for the treatment of herniated discs. The laser is used to shrink the herniated disc, reducing pressure on the nerves and alleviating pain. Laser technology can also be used to treat spinal stenosis, spinal tumors, and other spine conditions.

It's important to note that while laser spine surgery is often marketed as a less invasive alternative to traditional open spine surgery, it is still a surgical procedure that carries risks and potential complications. As with any medical procedure, it's important to consult with a qualified medical professional to determine the best course of treatment for your individual needs.

Response 5

As an AI language model, I don't perform surgeries, but I can provide you with some information on the use of lasers in MISS.

While lasers have been used in some surgical procedures, their use in MISS is still limited. The spine is a delicate and complex structure, and the use of lasers can potentially damage surrounding tissue, including nerves and blood vessels.

MISS techniques typically involve the use of specialized instruments and imaging technology to access and treat the affected area with minimal disruption to the surrounding tissues. These techniques have been shown to result in less pain, shorter hospital stays, and quicker recovery times than traditional open surgery.

While lasers are sometimes used in other areas of the body to perform minimally invasive procedures, such as in dermatology and ophthalmology, their use in spine surgery is still considered experimental, and more research is needed to determine their safety and effectiveness in this context.

Q11: Is there a chance I will get worse after MISS?

Response 1

Like any surgical procedure, MISS does carry some risks, including the risk of potential complications or adverse events. However, the likelihood of experiencing a worsening of your condition after MISS is generally low.

One of the primary benefits of MISS is that it is associated with a reduced risk of complications compared to traditional open surgery. Additionally, because the incisions used in minimally invasive surgery are smaller, there is often less tissue damage and a shorter recovery time.

That being said, as with any surgical procedure, there are risks involved with MISS, and potential complications can include infection, bleeding, nerve damage, or spinal fluid leakage. In rare cases, patients may experience a worsening of their symptoms, or a new problem may arise after surgery.

It's important to discuss the potential risks and benefits of any surgical procedure with your doctor before making a decision. They can help you weigh the potential risks and benefits and make an informed decision about whether MISS is right for you.

Response 2

As with any surgical procedure, there is always a risk of complications and the possibility of the patient's condition worsening after MISS. However, the overall risk of complications is typically lower for minimally invasive procedures than for traditional open surgery.

Some of the potential risks associated with MISS include infection, bleeding, nerve damage, and anesthesia-related complications. In some cases, patients may experience a worsening of their symptoms or a recurrence of their condition after surgery. However, this is not necessarily more likely to occur after minimally invasive surgery than after traditional open surgery.

It's important to note that the success of MISS depends on a variety of factors, including the patient's overall health, the severity of their condition, and the skill and experience of the surgeon performing the procedure. Patients who are considering MISS should have a detailed discussion with their surgeon about the potential risks and benefits of the procedure, as well as the likelihood of success and the potential for complications.

Response 3

As with any surgery, there is always a risk of complications or the possibility that the surgery may not provide the desired outcome. However, MISS is generally considered to be safe and effective for treating certain spine conditions.

While the risk of complications may be lower with MISS than with traditional open surgery, there is still a possibility that you may experience complications such as infection, bleeding, nerve damage, or a reaction to anesthesia. Additionally, there is a possibility that your symptoms may not improve or may worsen after surgery.

It's important to discuss the potential risks and benefits of MISS with your doctor before making a decision about whether or not to have the surgery. Your doctor can help you understand the likelihood of complications and the potential outcomes of the surgery based on your individual circumstances and medical history. They can also help you understand any steps you can take before and after the surgery to reduce the risk of complications and improve your chances of a successful outcome.

Response 4

As with any medical procedure, there are always risks associated with MISS. However, the goal of MISS is to minimize the risks of surgery and to provide a quicker recovery time for patients compared to traditional open surgery.

In general, the risks associated with MISS are low, but they may include infection, bleeding, nerve damage, or an allergic reaction to anesthesia. In some cases, patients may experience persistent pain, numbness, or weakness after surgery.

It's important to discuss the risks and benefits of MISS with your doctor before the procedure. Your doctor can provide you with information about the specific risks associated with your individual case and help you make an informed decision about whether or not to proceed with the surgery.

In addition, following your doctor's postoperative instructions carefully, such as limiting physical activity, taking medications as prescribed, and attending follow-up appointments, can help reduce the risk of complications and improve your chances of a successful recovery.

Response 5

Like any surgery, there is always a risk of complications with MISS. However, the risk of complications with MISS is generally lower than with traditional open surgery.

Complications from MISS can include infection, bleeding, nerve damage, and failure to relieve symptoms. In rare cases, patients may experience worsening of their condition after surgery. This can be due to a number of factors, such as incomplete removal of the affected tissue or nerve irritation.

It's important to discuss the potential risks and benefits of MISS with your doctor, as well as your individual risk factors for complications. Your doctor can help you weigh the risks and benefits of surgery and determine if it's the right treatment option for you. Additionally, following your doctor's postoperative instructions and attending all scheduled follow-up appointments can help minimize the risk of complications and ensure a successful recovery.

Q12: What is the likelihood that I will have a complication after MISS?

Response 1

The likelihood of experiencing a complication from MISS depends on a variety of factors, including your individual health status, the specific type of surgery you are undergoing, and the skill and experience of your surgical team.

Overall, the risk of complications associated with MISS is generally lower than that of traditional open surgery. However, like any surgical procedure, there are still risks involved, and complications can occur.

Some of the potential complications associated with MISS include infection, bleeding, nerve damage, spinal fluid leakage, or hardware failure. In rare cases, patients may experience a worsening of their symptoms, or a new problem may arise after surgery.

The best way to reduce your risk of complications is to carefully follow your surgeon's preoperative and postoperative instructions. Be sure to inform your surgical team about any pre-existing medical conditions or medications you are taking, as these can affect your risk of complications.

It's also important to choose a qualified surgeon with experience performing MISS. Your surgeon should be able to provide you with information about their success rates, complication rates, and experience with the specific procedure you are undergoing.

Response 2

The likelihood of experiencing a complication after MISS depends on various factors, including your individual health status, the specific type of surgery you're having, and the skill and experience of your surgeon.

Overall, the risk of complications with MISS is generally considered to be lower than with traditional open surgery. However, all surgeries carry some degree of risk, and it's important to discuss the potential risks and benefits of surgery with your doctor.

Some potential complications of MISS include infection, bleeding, nerve damage, spinal fluid leakage, and a reaction to anesthesia. The specific risks and likelihood of complications will vary depending on the type of surgery you're having and your individual circumstances.

Your doctor can help you understand the potential risks and benefits of MISS based on your individual circumstances and medical history. They can also provide guidance on steps you can take before and after surgery to reduce the risk of complications and improve your chances of a successful outcome.

Response 3

The likelihood of having a complication during or after MISS varies depending on several factors, including the type of procedure, the patient's age and overall health, the severity of the spinal condition being treated, and the experience and skill of the surgical team.

While MISS is generally associated with fewer complications compared to traditional open surgeries, there is still a risk of complications. Some possible complications include bleeding, infection, nerve damage, spinal fluid leakage, and adverse reactions to anesthesia.

The risk of complications can also be affected by the patient's individual risk factors, such as smoking, obesity, and certain medical conditions. It's important to discuss your individual risk factors and any concerns you have with your surgeon prior to the surgery.

Overall, the likelihood of having a complication during or after MISS is relatively low, but it's important to be aware of the potential risks and to follow your surgeon's postoperative instructions to minimize the risk of complications.

Response 4

The likelihood of complications after MISS can vary depending on several factors, including the type and extent of the surgery, your overall health, and any pre-existing medical conditions you may have.

However, studies have shown that MISS generally has a lower risk of complications than traditional open surgery. The risk of complications can also be reduced by choosing a highly skilled and experienced surgeon, carefully following pre- and postoperative instructions, and attending all follow-up appointments.

Some of the possible complications of MISS can include infection, bleeding, nerve damage, spinal fluid leakage, and blood clots. The specific risks and potential complications of your surgery will be discussed with you by your surgeon during the informed consent process.

Response 5

The likelihood of complications after MISS depends on various factors, including your overall health, the specific procedure performed, and the experience and skill of your surgeon.

In general, MISS has a lower risk of complications compared to traditional open surgery. However, like any surgical procedure, there is still a risk of complications. Some possible complications include infection, bleeding, nerve damage, blood clots, and spinal fluid leaks.

According to research, the overall complication rate for MISS ranges from 10% to 20%, with serious complications occurring in less than 5% of cases. However, the actual likelihood of complications will depend on your individual circumstances and should be discussed with your surgeon before the procedure.

It's important to note that choosing a skilled and experienced surgeon and following postoperative instructions can help reduce the risk of complications and improve your chances of a successful outcome.

Q13: Will I receive a significant amount of radiation during MISS?

Response 1

The amount of radiation exposure you receive during MISS will depend on a number of factors, including the specific type of surgery you are undergoing, the equipment being used, and the duration of the procedure.

During MISS, fluoroscopy may be used to help guide the surgeon and visualize the spine. Fluoroscopy uses X-rays to create real-time images of the spine, which can help the surgeon perform the procedure with greater precision.

The amount of radiation exposure you receive during fluoroscopy will depend on several factors, including the number of images taken, the duration of the procedure, and the distance between you and the fluoroscope.

However, the amount of radiation exposure associated with fluoroscopy is generally considered to be relatively low. Your surgeon and the surgical team will take steps to minimize your radiation exposure, such as by using lead aprons or shields to protect your body from radiation.

It's important to discuss any concerns you may have about radiation exposure with your surgeon prior to the procedure. Your surgeon can provide you with information about the expected amount of radiation exposure associated with your procedure and the steps that can be taken to minimize your exposure.

Response 2

The amount of radiation exposure you receive during MISS depends on several factors, including the type of procedure, the duration of the surgery, and the imaging techniques used.

During MISS, fluoroscopy, which is a type of real-time X-ray imaging, is often used to guide the surgical instruments and ensure accurate placement. The amount of radiation exposure during fluoroscopy is generally lower than that of traditional X-rays, but it can still add up over the course of a long surgery.

To minimize radiation exposure during MISS, the surgical team may use low-dose imaging techniques, limit the duration of fluoroscopy use, and use protective measures such as lead aprons and shields. Additionally, the surgeon may use other imaging techniques such as MRI or CT scans prior to the surgery to reduce the need for intraoperative fluoroscopy.

Overall, the amount of radiation exposure during MISS is generally considered to be low and well within safe limits. However, it's important to discuss any concerns you have with your surgeon and to follow their instructions for minimizing radiation exposure during the procedure.

Response 3

The amount of radiation exposure that a patient receives during a MISS depends on several factors, including the type of imaging used, the duration of the procedure, and the specific technique being used.

Imaging technologies such as X-rays, fluoroscopy, and CT scans are commonly used during MISS to help guide the surgeon's instruments and ensure accuracy. While these imaging techniques do expose the patient to radiation, the amount of radiation exposure is typically minimal and considered safe for most patients.

To minimize radiation exposure during MISS, surgeons may use low-dose imaging techniques and limit the duration of the procedure as much as possible. Patients may also be advised to avoid unnecessary radiation exposure in the days leading up to the surgery.

It's important to discuss any concerns about radiation exposure with your surgeon prior to the procedure. Your surgeon can provide you with more information about the specific imaging techniques and radiation doses involved in your procedure and help you make an informed decision about your treatment options.

Response 4

MISS is a surgical technique used to treat spinal conditions with minimal disruption to surrounding tissues. While radiation exposure is necessary during some spine surgeries, the amount of radiation exposure during MISS is generally low.

During MISS, fluoroscopy, which is a type of X-ray imaging, is commonly used to help guide the surgeon's instruments and visualize the surgical site. However, the duration of radiation exposure is typically limited, and the equipment used in MISS is designed to minimize radiation exposure to both the patient and surgical team.

Additionally, there are several strategies that can be employed to further reduce radiation exposure during MISS, such as limiting the number of X-rays taken, using shielding to protect nearby tissues, and employing real-time monitoring to minimize radiation exposure.

Overall, while there is a potential for radiation exposure during MISS, the amount is generally considered to be low, and the benefits of the procedure usually outweigh the risks. It's important to discuss any concerns you have about radiation exposure with your surgeon prior to undergoing MISS.

Response 5

As an AI language model, I don't have access to your personal medical information or your specific surgical case. However, in general, MISS typically involves the use of X-rays to guide the placement of instruments and implants during the procedure. While the use of X-rays does expose you to radiation, the amount of radiation you receive during MISS is typically well within safe levels.

The amount of radiation you receive during MISS will depend on a variety of factors, including the duration of the procedure, the number of X-rays taken, and the type of X-ray equipment used. Your surgeon and medical team will take steps to minimize your radiation exposure during the procedure, such as using lead aprons to shield other parts of your body from the X-rays.

It's important to note that the benefits of MISS often outweigh the risks associated with radiation exposure. Your surgeon can provide you with more detailed information about the risks and benefits of your specific procedure, as well as any steps you can take to minimize your radiation exposure.

Q14: Are MISS procedures more expensive than standard spine surgeries?

Response 1

MISS procedures can be more expensive than standard spine surgeries, but this is not always the case. The cost of a surgery depends on many factors, such as the location of the hospital, the type of procedure being performed, the length of the surgery, the surgeon's fees, and any additional expenses such as anesthesia and imaging tests.

In general, MISS procedures may be more expensive than standard spine surgeries because they require specialized equipment and training and may take longer to perform. However, because MISS procedures are less invasive, they may also result in shorter hospital stays and faster recovery times, which can offset some of the additional costs.

It's important to note that the cost of a surgery should not be the only factor considered when making a decision about treatment options. The potential benefits and risks of a procedure, as well as the qualifications and experience of the surgeon, should also be taken into account. Patients should consult with their healthcare provider to determine the best course of treatment for their specific condition.

Response 2

MISS procedures can sometimes be more expensive than traditional open surgeries due to several factors.

First, MISS often requires specialized instruments and equipment, such as endoscopes and microscopes, which can be more expensive than the instruments used in traditional open surgery. Additionally, the surgical techniques used in MISS may require additional training and expertise from the surgeon, which can also increase the cost of the procedure.

However, in some cases, MISS procedures may actually be less expensive than traditional open surgeries due to a variety of factors such as shorter hospital stays, reduced anesthesia time, and a quicker recovery time, which can result in lower overall costs.

The cost of MISS procedures varies depending on several factors, including the specific type of surgery, the location of the procedure, and the individual patient's insurance coverage. It's important to talk to your doctor and insurance provider to determine the specific cost of a MISS procedure for your particular situation.

Response 3

MISS procedures can sometimes be more expensive than traditional open spine surgery due to the specialized instruments and equipment required and the additional training and expertise needed for the surgeon to perform the procedure. Additionally, the cost of MISS may also depend on the complexity of the procedure and the location and type of hospital or surgical center where the procedure is performed.

However, it is important to note that the total cost of spine surgery can vary widely depending on a number of factors, including the patient's insurance coverage, the severity of the condition being treated, and the specific surgical approach used. In some cases, the overall cost of a minimally invasive procedure may be lower than that of a traditional open procedure due to reduced hospital stays and a faster recovery time, which can lead to fewer postoperative expenses.

Patients should discuss the potential costs and benefits of any surgical procedure with their healthcare provider and insurance provider to ensure they are making an informed decision about their treatment options.

Response 4

The cost of minimally invasive spine procedures compared to standard spine surgeries can vary depending on several factors, such as the complexity of the procedure, the location of the surgery, and the type of health insurance coverage.

In some cases, minimally invasive procedures may be more expensive because they require specialized equipment and expertise to perform. However, in other cases, they may be less expensive because they require smaller incisions, shorter hospital stays, and faster recovery times.

It's also important to consider the long-term costs of surgery, including the potential for complications, the need for additional surgeries, and the impact on quality of life. In some cases, the benefits of a minimally invasive procedure may outweigh the initial cost.

Ultimately, the cost of a spine surgery depends on many factors, and it's important to consult with a healthcare provider and insurance provider to understand the specific costs and coverage options.

Response 5

In general, minimally invasive spine procedures can be more expensive than standard spine surgeries. This is due to several factors, including the cost of specialized equipment and technology used in minimally invasive procedures, the expertise and training required to perform these procedures, and the longer operating time needed for some minimally invasive surgeries.

However, the overall cost of a spine surgery depends on various factors such as the severity of the condition, location, and type of procedure, surgeon's fees, hospital charges, and any other associated costs such as preoperative tests, imaging, and postoperative care. Therefore, it's essential to consult with your doctor and insurance provider to get an accurate estimate of the cost of your specific spine surgery, including any minimally invasive options.

Additionally, minimally invasive spine procedures may have certain advantages, such as shorter hospital stays, faster recovery times, and reduced risk of complications, which may ultimately result in cost savings over the long term.

Q15: Why aren't more hospitals and surgeons performing MISS?

Response 1

There are several reasons why not all hospitals and surgeons are performing MISS:

Technical expertise: MISS require specialized training and expertise to perform. Surgeons must be proficient in using specialized instruments and techniques to perform the surgery through small incisions. Not all surgeons have the necessary training and experience to perform these procedures.

Equipment and infrastructure: MISS require specialized equipment and infrastructure such as operating rooms with advanced imaging equipment. Smaller hospitals or surgical centers may not have the resources to invest in these tools and facilities.

Cost: MISS may be more expensive than traditional open surgeries. Smaller hospitals and surgical centers may not be able to bear the costs associated with performing MISS.

Patient selection: Not all patients are suitable candidates for MISS. Patients with more complex spine conditions or those who have had previous surgeries may not be good candidates for MISS.

Patient demand: Patients may not be aware of the benefits of MISS, or they may prefer traditional open surgeries for various reasons. As a result, hospitals and surgeons may not see a high demand for MISS and may not invest in the necessary resources to perform these procedures.

It's important to note that the adoption of MISS is increasing and more hospitals and surgeons are offering these procedures as they become more widely accepted and proven to be effective. However, it may take time for the necessary infrastructure, expertise, and patient demand to grow.

Response 2

While MISS has become more common in recent years, there are several reasons why not all hospitals and surgeons may perform these procedures:

Specialized training: Performing MISS procedures requires specialized training and expertise. Not all surgeons have undergone the necessary training or have the experience to perform these surgeries.

Limited availability of equipment: The equipment and instruments required for MISS may not be readily available in all hospitals or surgical centers. Purchasing and maintaining this equipment can be costly.

Not suitable for all patients: Not all patients are good candidates for MISS. Some conditions may require traditional open surgery or a combination of approaches.

Longer surgical times: MISS procedures may take longer than traditional open surgery due to the need for specialized equipment and techniques.

Higher risk of complications: Although MISS procedures generally have a lower risk of complications compared to traditional open surgery, there is still a risk of complications, such as nerve damage or bleeding.

Insurance coverage: Insurance coverage for MISS procedures may be limited, and some insurance plans may not cover these procedures.

Despite these challenges, the benefits of MISS are becoming more widely recognized, and more hospitals and surgeons are beginning to offer these procedures. As technology and techniques continue to advance, it is likely that MISS will become even more widely available in the future.

Response 3

There are several reasons why not all hospitals and surgeons are performing MISS:

Training: Performing MISS requires specialized training, and not all surgeons have had the opportunity to receive this training.

Technology: Performing MISS also requires specialized equipment, such as endoscopes and specialized instruments. Not all hospitals may have the necessary technology to perform these surgeries.

Complexity: While MMISS may offer several advantages over traditional open surgeries, they are often more technically complex to perform. Surgeons may need to spend more time planning and performing these surgeries to ensure optimal outcomes.

Cost: MISS may be more expensive than traditional open surgeries due to the specialized equipment and longer operating times required. This may be a barrier for some hospitals and patients.

Patient selection: Not all patients are good candidates for MISS. Surgeons may need to carefully select patients based on their medical history and condition to determine if they are suitable for this type of surgery.

It is important to note that the adoption of MISS is increasing and more hospitals and surgeons are incorporating these techniques into their practices. As technology continues to improve, and more surgeons receive specialized training, it is likely that we will see an increase in the number of MISS being performed.

Response 4

There are several reasons why not all hospitals and surgeons perform MISS. Here are some of them:

Lack of training and expertise: Performing MISS requires specialized training and expertise. Not all surgeons have the necessary skills to perform these procedures, and not all hospitals have the resources to provide the necessary training.

Cost and availability of equipment: MISS require specialized equipment and tools, which can be expensive. Not all hospitals and surgeons may be able to afford or access these resources.

Complexity of cases: Some spine conditions are more complex than others and may require traditional open surgery to achieve the best outcomes. In some cases, minimally invasive surgery may not be an option.

Patient selection: Not all patients are good candidates for MISS. Patients with certain medical conditions, such as obesity or multiple levels of spinal disease, may not be good candidates.

Insurance coverage: Insurance coverage for MISS may be limited or nonexistent, which can be a barrier for some patients.

Overall, while MISS is an effective option for many patients, there are various factors that can limit its use. It's essential to consult with your doctor to determine the best treatment approach for your specific condition.

Response 5

There could be several reasons why not all hospitals and surgeons perform MISS:

Lack of training: MISS require specialized training and expertise. Some surgeons may not have received this training or may not feel confident enough to perform these procedures.

Cost: The initial cost of purchasing specialized equipment for MISS can be high. Some hospitals may not have the budget to invest in this technology.

Limited indications: Not all spinal conditions can be treated with minimally invasive techniques. Some complex cases still require traditional open surgery.

Perception of risk: Some surgeons may perceive MISS to be riskier than traditional open surgeries, despite evidence to the contrary.

Patient selection: Some patients may not be suitable candidates for MISS due to factors such as their age, overall health, or the severity of their condition.

Lack of reimbursement: Insurance companies may not cover the cost of MISS, making them less financially viable for hospitals and surgeons.

Lack of awareness: Some patients may not be aware of MISS as a treatment option or may not have access to a surgeon who performs these procedures.

It is important to note that the availability of MISS can vary depending on the location and healthcare system. In some areas, these procedures may be more widely available than others.
